# Timed Up and Go test score and factors associated with a moderate-to-high risk of future falls in patients scheduled for vascular surgeries—a cross-sectional study

**DOI:** 10.3389/fpubh.2024.1363828

**Published:** 2024-03-21

**Authors:** Renata Piotrkowska, Wioletta Anna Mędrzycka-Dąbrowska, Lucyna Tomaszek

**Affiliations:** ^1^Department of Surgical Nursing, Medical University of Gdansk, Gdańsk, Poland; ^2^Clinic of Cardiac and Vascular Surgery, University Clinical Center, Gdańsk, Poland; ^3^Department of Anesthesiology Nursing and Intensive Care, Medical University of Gdańsk, Gdańsk, Poland; ^4^Department of Specialist Nursing, Faculty of Medicine and Health Sciences, Kraków Academy of Andrzej Frycz Modrzewski, Kraków, Poland; ^5^Institute of Tuberculosis and Lung Diseases, Rabka-Zdrój Branch, Rabka-Zdrój, Poland

**Keywords:** falls, vascular surgeries, risk factors, perioperative care, injury

## Abstract

**Introduction:**

Peripheral artery and aorta diseases contribute to complex consequences in various areas, as well as increasing physical and mental discomfort resulting from the progressive limitation or loss of functional capacities, in particular in relation to walking, decreased endurance during physical exercise, a drop in effort tolerance, and pain suffered by patients. Limitations in functional capacities also increase the risk of falls. Most falls take place during the performance of simple activities. The aim of this study was to investigate factors associated with moderate-to-high risk of future falls in patients scheduled for vascular surgeries.

**Methods:**

This cross-sectional study included patients aged 33–87, scheduled for vascular surgeries. Based on the Timed Up and Go test, patients were categorized as having a moderate-to-high (≥ 10 s) or low risk of falls. Multiple logistic regression was carried out to assess the relationship between fall-risk levels and independent sociodemographic and clinical variables.

**Results:**

Forty-eight percent of patients were categorized as having a moderate-to-high risk of future falls. Females (OR = 1.67; Cl95%: 1.07–2.60) and patients who suffered from hypertension (OR = 2.54; Cl95%: 1.19–5.40) were associated with a moderate-to-high risk of future falls. The Barthel Index correlated negatively (OR = 0.69; Cl95%: 0.59–0.80), while age correlated positively with fall-risk levels (OR = 1.07; Cl95%: 1.02–1.12).

**Conclusion:**

Factors that may be associated with a moderate-to-high risk of future falls in patients scheduled for vascular surgeries include age, female gender, hypertension, and the Barthel Index.

## 1 Introduction

In recent years, morbidity and mortality caused by peripheral artery and aorta diseases have increased (1, 2). Those diseases contribute to complex consequences in various areas, as well as increasing physical and mental discomfort as a result of the progressive limitation or loss of functional capacities, in particular in relation to walking, decreased endurance during physical exercise, a drop in effort tolerance, and pain suffered by patients. As the disease develops, changes taking place in the human organism can initially contribute to a slight reduction in functional capacities, which can subsequently result in complete dependence on carers. Limitations in functional capacities also increase the risk of falls (3, 4). As defined by the World Health Organization (WHO), a fall is defined as an event, which results in a person coming to rest inadvertently on the ground or floor or other lower level usually as a result of the simultaneous occurrence of many unfavorable factors. There are usually multiple causes of falls, which result from interactions between predisposing chronic diseases. Most falls take place during the performance of simple activities such as getting up, sitting down, bending, and walking (5).

The British National Institute for Health and Clinical Excellence (NICE) reports that there are up to 400 fall risk factors (6). Not all of them can be influenced or prevented. It must be underlined, however, that our society must develop a strategy for reducing the risk of falls.

Previous prospective cohort studies identified such fall risk factors as falls reported by patients (7, 8), lower limb weakness (7, 8), old age (9–11), female gender (10), cognitive function disorders (9, 10), balance disorders (10), use of psychotropic drugs (10), arthritis (10), stroke (11), orthostatic hypotension (10), dizziness (10), fainting (12), and nocturia (13, 14).

### 1.1 Fall prevention

By diagnosing the reasons for falls, it is possible to implement actions aimed at minimizing the frequency of falls. In hospital conditions, it is very important to identify patients with a high risk of falls at check-in in order to prevent falls (6).

This should be a multi-dimensional process aimed at identifying deficits in all operating zones of a human being. In clinical conditions, effective intervention aimed at fall prevention includes the assessment and inclusion of individual fall risk factors, and an individual approach is recommended in the best practices of the American and British Geriatric Society (15). In addition, as falls of hospitalized patients can result in medical litigations (16), it is necessary to take measures to prevent falls at hospitals.

Fall prevention measures should be implemented in four basic areas: health education, the overall assessment of fall risk factors, the modification of environmental risk factors, and the implementation of individually prepared rehabilitation programs. Knowledge of the frequency of falls and related factors provides important information to managers taking care of hospitalized patients. Throughout the world, nursing leaders support the implementation of surgery-related programs aimed at improving the functional fitness of patients (17).

The purpose of the study was to assess the risk of falls in patients with peripheral artery and aorta diseases, as well as the impact of sociodemographic and clinical variables on the risk of future falls.

## 2 Materials and methods

### 2.1 Study design, setting

It was a cross-sectional study including 100 patients undergoing vascular surgery. The study was conducted between November 2022 and September 2023 in the Department of Cardiac Surgery and Vascular Surgery of the University Clinical Center in the north of Poland. The guidelines of Strengthening the Reporting of Observational Studies in Epidemiology (STROBE) (18) were followed.

### 2.2 Participants

The studied group included patients aged 33–87 of both sexes, with abdominal aortic aneurysm (AAA), peripheral arterial disease (PAD), or carotid artery disease (CAD). The reasons for exclusion were lack of qualification for a vascular surgery and lack of an informed consent form. The patients were not gratified in any way and gave their voluntary consent to take part in the study.

### 2.3 Data collection

Sociodemographic and clinical data were collected. The former included variables such as age, gender, marital status, place of residence, education level, professional activity, and type of work performed currently or in the past. Clinical data included Timed Up and Go (TUG), body weight, body height, Barthel Index, primary disease, comorbidities, accompanying symptoms, number of medications taken at one time, wounds or skin damage, and pain.

Timed Up and Go is a commonly applied screening tool that helps to identify patients that are likely to fall. The test is conducted in accordance with the following rules: at first, the tested person sits on a chair with their back on the backrest; then, on the command “START,” they get up and walk 3 m (on a level floor at a normal pace); next, having crossed a line, they take a 180-degree turn and come back to the chair to sit down again. The time is measured by a researcher with a stopwatch when the patient is instructed on how the test should be done. The test was performed in a medical examination room located in the clinic on the first day of hospitalization. A test result <10 s indicates a low risk of falls (correct functional fitness), 10–19 s—a moderate risk of falls (the tested person can go outdoors on their own without walking support equipment and are independent in most of their daily living activities), and ≥ 19—a high risk of falls (significantly limited functional fitness, the tested person cannot go outdoors on their own and are recommended to use walking support equipment) (19). Finally, for the purposes of this study, patients were categorized as having a low (TUG < 10 s) or moderate-to-high risk of falls (TUG ≥ 10 s).

The Numerical Rating Scale (NRS) provides for 11 pain intensity degrees, i.e., from 0 to 10, where 0 means a lack of pain and 10 the worst imaginable pain. The scale is characterized by a significant repetitiveness of results and is useful in scientific studies (20).

The Barthel Index is an international questionnaire assessing a patient’s fitness. It is one of the tools assessing basic activities of daily living (ADLs). The Barthel Index consists of 10 questions analyzing the patient’s capacity to perform basic ADLs. The patient can obtain from 0 to 100 points in total. The greater the score, the more self-reliant the patient is. There are three groups of scores: 0–20 points: the person is not self-reliant, heavy condition; 25–85 points: the person needs partial help, moderately heavy condition; and 90–100 points: the person is self-reliant, light condition (21).

The original questionnaire enabled us to collect social and demographic data, such as sex, age, education level, place of residence, marital status, and professional activeness. The questions included the identification of fall risk factors, the history of diseases, the reason for hospitalization, disease symptoms, and medicines taken.

### 2.4 Outcomes

The primary outcomes described fall-risk levels in patients scheduled for vascular surgeries. The secondary outcomes included sociodemographic and clinical factors associated with a moderate-to-high risk of future falls.

### 2.5 Statistical analysis

Intergroup differences (moderate-to-high fall risk vs. low fall risk) for categorical data were assessed using the Chi-square or Fisher’s test. These data were shown as absolute numbers and percentages. The Mann–Whitney test was used for continuous variables, which were presented as medians, and upper and lower quartiles. Data distribution was tested with the Shapiro–Wilk test.

Multiple logistic regression was implemented to assess the relationship between the dependent variable “Fall-Risk Levels” (moderate-to-high vs. low) and the independent sociodemographic and clinical variables.

A simple logistic analysis was firstly performed to select the predictors—a variable with a *p* value <0.1 was entered into the multiple regression model. The backward elimination technique was used to build an effective model. The Hosmer-Lemeshow test of goodness of fit suggests the model is a good fit to the data as *p* > 0.05. Nagelkerke’s *R*^2^ describes the proportion of variance in the outcome that the model successfully explains. The significance of individual coefficients in the model was tested by use of Wald statistics. The odds ratio was also calculated with a 95% confidence interval.

Logistic regression can be applied and provided that the sample is large enough (*n*), i.e., *n* should be greater than 10 × (*k* + 1), where *k* is the number of independent variables. In this study, the conditions were met because *n* = 100, which means that it is greater than *n* calculated on the basis of the above formula [10 × (8 + 1)] = 90 (22).

All calculations were performed using STATISTICA v.13.3. (TIBCO Software Inc., 2017, Krakow, Poland). Values of *p* < 0.05 were considered significant for all statistical analyses.

### 2.6 Ethical considerations

While collecting the data, the ethical principles set out in the Helsinki Declaration were taken into account. The research was approved by the Independent Bioethical Committee at the Medical University of Gdańsk, number NKBBN/348/2022.

## 3 Results

**Figure 1:**
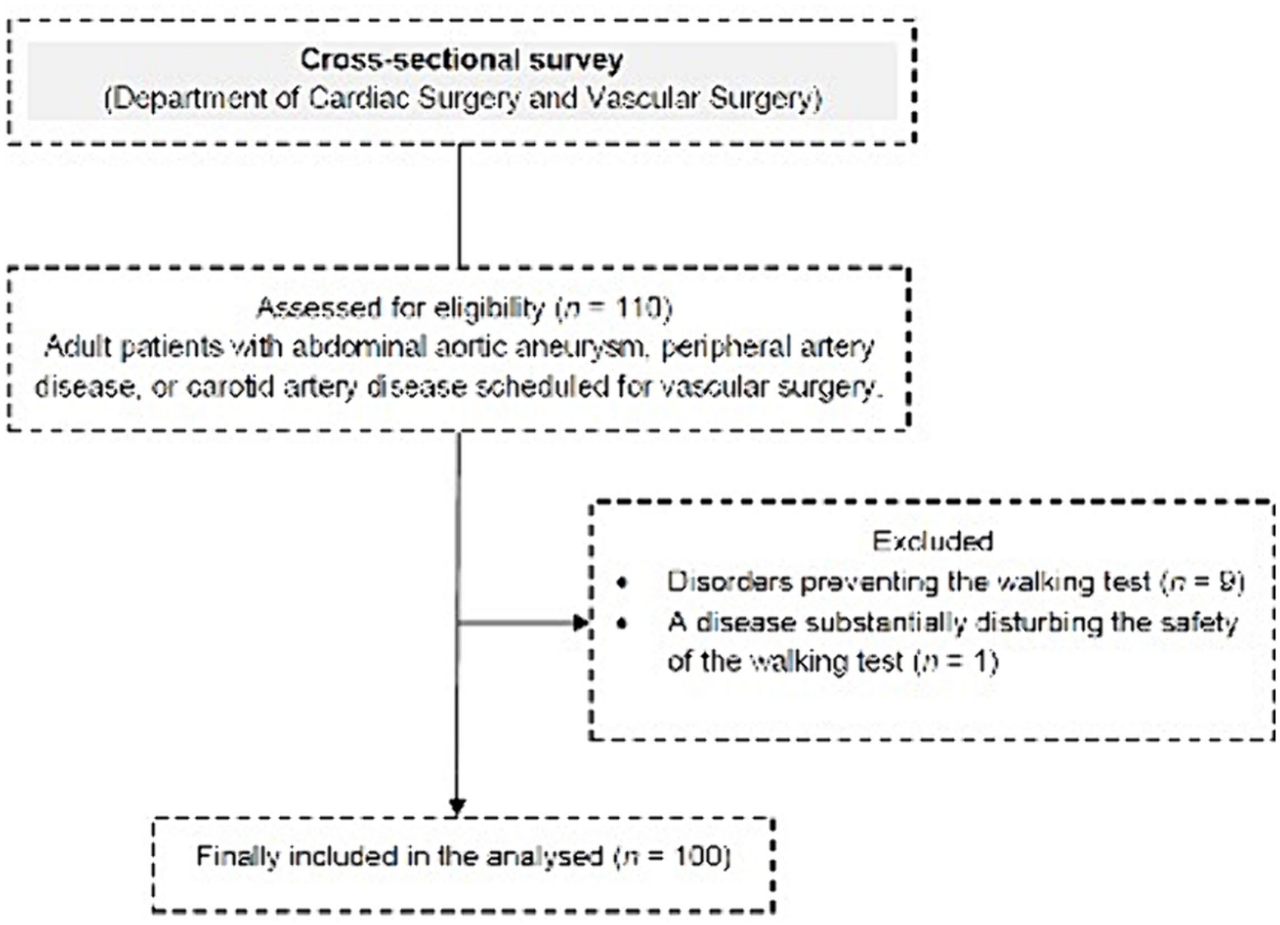
Flow chart of the study design.

The study included 100 patients. The flow chart of the study design is presented in Figure 1.

### 3.1 Sociodemographic characteristics of the patients

The detailed sociodemographic characteristics of the patients are presented in Table 1. Forty-eight percent of the study group was categorized as having a moderate-to-high fall risk. The median age in this group was significantly higher compared to the median age in the low fall risk group (71 vs. 68 years; *p* = 0.008). Gender and marital status were found to be significantly different between the groups (*p* < 0.05). The distribution of other sociodemographic variables is similar in both groups—patients were mostly urban residents (83%), married (66%), and had a similar level of education (primary school—18%, vocational education—32.0%, high school—30.0%, and university—20%). The vast majority of patients currently did not run any professional activity (82%)—they declared that a white-collar job is/was the dominant type of work performed by them currently or in the past (65%). It should be emphasized that currently every fourth hospitalized patient has smoked cigarettes for at least 18 years (maximum 50 years), and 61% of them smoked in the past. Alcohol consumption more than once a month was declared by 30% of the patients.

**Table 1:** Sociodemographic characteristics of the patients.

Variables	Fall-risk levels		Statistics values	*p* value

	Moderate-to-high (*n* = 48)	Low (*n* = 52)		
Age (years)	71 [61; 79]	68 [60; 73]	*Z* = −2.66	0.008
Gender				
Female	23 (47.9)	13 (25.0)	*χ^2^ =* 5.69	0.02
Male	25 (52.1)	39 (75.0)		
Married/domestic partnership				
Yes	26 (54.2)	40 (76.9)	*χ^2^ =* 5.76	0.02
No	22 (45.8)	12 (23.1)		
Place of residence				
City	39 (81.2)	44 (84.6)	*χ^2^ =* 0.20	0.65
Village	9 (18.7)	8 (15.3)		
Education level				
High school/University	22 (45.8)	28 (53.8)	*χ^2^ =* 0.64	0.42
Primary school/Vocational education	26 (54.2)	24 (46.1)		
Professional activity				
Yes	5 (10.4)	13 (25.0)	*χ^2^ =* 2.67	0.07
No	43 (89.5)	39 (75.0)		
Type of work performed currently or in the past				
Blue-collar job	32 (66.7)	33 (63.5)	*χ^2^ =* 0.11	0.73
White-collar job	16 (33.3)	19 (36.5)		
Smoking	11 (44.0)	14 (56)	*χ^2^ =* 0.21	0.64
Alcohol consumption more than once a month	12 (25.0)	18 (34.6)	*χ^2^ =* 1.10	0.29

Age is presented as medians [upper and lower quartiles], and categorical variables as absolute numbers (percentages).

### 3.2 Clinical characteristics of the patients

The clinical characteristics of the patients are shown in Table 2. The most common indication for surgery was peripheral arterial disease (57%). Among the comorbidities, patients most often indicated hypertension, the frequency of which was higher in the moderate-to-high risk of falls’ group than in the low risk of falls’ group (89.6 vs. 69.2%; *p* = 0.01). Patients with a moderate-to-high risk of falls also had a higher incidence of pain (60.4 vs. 40.4%; *p* = 0.04), especially in the lower limbs at rest (66.7 vs. 42.3%; *p* = 0.01) and intermittent claudication (50.0 vs. 26.9%; *p* = 0.02). In this group, more incidents were observed of balance disorders/falls (43.7 vs. 15.4%; *p* = 0.0002), wounds or skin damage (35.4 vs. 11.5%; *p* = 0.004), and patients taking more than 5 medications at one time (64.6 vs. 40.4%; *p* = 0.01). In addition, the significant intergroup differences were noted in medians of clinical variables such as: the Barthel Index (90 [85; 95] vs. 100 [97; 100]; *p* < 0.0001), the sum of comorbidities (3.5 [2; 4] vs. 3 [2; 4]; *p* = 0.04), and the sum of accompanying symptoms (3 [2; 3] vs. 2 [1; 2]; *p* = 0.0002).

**Table 2:** Clinical characteristics of the patients.

Variables	Fall-risk levels		Statistics values	*p* value

	Moderate-to-high (*n* = 48)	Low (*n* = 52)		
				
Timed up and go	14 [12.0; 18.7]	8.5 [7.7; 9.0]	*Z* = −5.24	< 0.001
Body weight (kg)	75 [65; 85]	75 [65; 86]	*Z* = 0.08	0.93
Body height (cm)	168 [164; 173]	170 [165; 176]	*Z* = 1.83	0.06
Barthel index	90 [85; 95]	100 [97; 100]	*Z* = 6.45	< 0.001
Duration of primary disease (month)	12 [3; 42]	24 [5; 60]	*Z* = 1.48	0.14
Sum of comorbidities	3.5 [2; 4]	3 [2; 4]	*Z* = −2.02	0.04
Sum of accompanying symptoms	3 [2; 3]	2 [1; 2]	*Z* = −3.77	0.0002
Primary disease				
Peripheral arterial disease	35 (72.9)	22 (42.3)	*χ^2^ =* 11.31	0.003
Carotid artery disease	6 (12.5)	20 (38.5)		
Abdominal aortic aneurysm	7 (14.6)	10 (19.2)		
Comorbidities				
Diabetes	22 (45.8)	16 (30.8)	*χ^2^ =* 2.40	0.12
Hypertension	43 (89.6)	36 (69.2)	*χ^2^ =* 5.06	0.01
Atherosclerosis	38 (79.2)	38 (73.1)	*χ^2^ =* 0.51	0.48
Ischemic stroke	9 (18.7)	9 (17.3)	*χ^2^* = 0.03	0.85
Asthma	3 (6.2)	4 (7.7)	*χ^2^ =* 0.01	1.00
Chronic obstructive pulmonary disease	5 (10.4)	7 (13.5)	*χ^2^ =* 0.02	0.76
Rheumatoid arthritis	5 (10.4)	1 (1.9)	*χ^2^ =* 1.86	0.10
Heart failure	14 (29.2)	11 (21.1)	*χ^2^ =* 0.85	0.35
Kidney failure	3 (6.2)	2 (3.8)	*χ^2^ =* 0.008	0.67
Accompanying symptoms				
Pain in lower limbs at rest	32 (66.7)	22 (42.3)	*χ^2^ =* 5.96	0.01
Spine pain	9 (18.7)	7 (13.5)	*χ^2^ =* 0.52	0.47
Abdomen pain	3 (6.2)	6 (11.5)	*χ^2^ =* 0.32	0.49
Intermittent claudication	24 (50.0)	14 (26.9)	*χ^2^ =* 5.64	0.02
Dizziness	11 (22.9)	14 (26.9)	*χ^2^ =* 0.21	0.64
Fainting	1 (2.0)	4 (7.7)	*χ^2^ =* 0.68	0.36
Blurred vision	8 (16.7)	8 (15.4)	*χ^2^ =* 0.03	0.86
Incidents of balance disorders/falls	21 (43.7)	8 (15.4)	*χ^2^ =* 9.75	0.002
Number of medications taken at one time				
≤ 5	17 (35.4)	31 (59.6)	*χ^2^ =* 5.85	0.01
> 5	31 (64.6)	21 (40.4)		
Wounds or skin damage	17 (35.4)	6 (11.5)	*χ^2^ =* 8.03	0.004
Pain	29 (60.4)	21 (40.4)	*χ^2^ =* 4.00	0.04
Numerical rating scale (0–10)	4 [3; 6]	3 [1; 6]	*Z* = −1.11	0.26

Continuous variables are presented as medians (upper and lower quartiles), and categorical variables as absolute numbers (percentages).

### 3.3 Sociodemographic factors associated with a moderate-to-high risk of future falls

Sociodemographic factors associated with a moderate-to-high fall risk are presented in Table 3. Females have a higher likelihood of future falls than males (OR = 1.67; Cl95%: 1.07–2.60). Age correlates positively with fall-risk levels (OR = 1.07; Cl95%: 1.02–1.12).

**Table 3:** Sociodemographic factors associated with a moderate-to-high risk of future falls.

Variables	*B*	SE *(B)*	Wald test	*p*	*OR (Cl 95%)*
Simple logistic regression					
Age	0.07	0.02	7.54	0.006	1.07 (1.02 to 1.12)
Female ^Reference: Male^	0.51	0.22	5.54	0.02	1.66 (1.09 to 2.53)
Married / domestic partnership ^Reference: No^	0.52	0.22	5.59	0.02	1.68 (1.09 to 2.58)
Professional activity ^Reference: No^	0.52	0.28	3.40	0.06	1.69 (0.97 to 2.96)
Multiple logistic regression model					
Age	0.07	0.02	7.17	0.007	1.07 (1.02 to 1.12)
Female ^Reference: Male^	0.51	0.23	5.14	0.02	1.67 (1.07 to 2.60)

B, Regression coefficient; SE, Standard error; OR, Odds ratio; and CI, Confidence interval.

Multivariable logistic regression model: *R*^2^ Nagelkerke = 0.18; Hosmer-Lemeshow = 5.67; *p* = 0.68.

### 3.4 Clinical factors associated with a moderate-to-high risk of future falls

Clinical factors associated with a moderate-to-high fall risk are shown in Table 4. Patients suffering from hypertension have a 2.54 times higher risk of future unintentional falls than those without such diseases (OR = 2.54; Cl95%: 1.19–5.40). The Barthel Index correlates negatively with the fall-risk levels (OR = 0.69; Cl95%: 0.59–0.80).

**Table 4:** Clinical factors associated with a moderate-to-high risk of future falls.

Variables	*B*	SE *(B)*	Wald test	*p*	*OR (Cl 95%)*
Simple logistic regression					
Hypertension ^Reference: No^	0.67	0.28	3.09	0.08	1.95 (1.13—3.38)
Barthel index	−0.34	0.07	25.93	<0.001	0.71 (0.63–0.81)
Pain in lower limbs at rest ^Reference: No^	0.52	0.21	5.83	0.01	1.65 (0.09–2.48)
Intermittent claudication ^Reference: No^	0.50	0.21	5.51	0.02	1.65 (1.09–2.50)
Incidents of balance disorders/falls ^Reference: No^	0.72	0.24	9.09	0.003	2.07 (1.29–3.32)
Number of medications >5 ^Reference: ≤ 5^	0.49	0.21	5.74	0.02	1.64 (1.09–2.46)
Wounds or skin damage ^Reference: No^	0.72	0.26	7.38	0.007	2.05 (1.22–3.44)
Pain ^Reference: No^	−0.41	0.20	3.95	0.047	0.67 (0.45–0.99)
Multiple logistic regression model					
Hypertension ^Reference: No^	0.93	0.39	5.82	0.02	2.54 (1.19–5.40)
Barthel index	−0.38	0.08	24.13	<0.001	0.69 (0.59–0.80)

B, Regression coefficient; SE, Standard error; OR, Odds ratio; and CI, Confidence interval.

Multivariable logistic regression model: R2 Nagelkerke = 0.57; Hosmer-Lemeshow = 0.90; and *p* = 0.92.

## 4 Discussion

The purpose of this study was to assess the fall risk factors for patients subject to vascular procedures in hospital, as well as the impact of sociodemographic and clinical variables on the risk of future falls. The analysis covered patients with PAD, AAA, and CAD. This is a group of diseases with a common origin connected with the development of atherosclerosis (1–3). The results of this study revealed that age, female gender, hypertension, and the Barthel Index were factors determining the moderate-to-high risk of falls in patients scheduled for vascular surgeries.

In the Polish literature, there are no works concerning the risk of falls in patients subject to vascular procedures. From the point of view of nursing, the knowledge of risk factors and conditions in which falls are most frequent is of basic importance because it enables the threat to be predicted and preventive measures taken. The National Institute for Health and Care Excellence in England recommends that patients of 65 and older that are hospitalized due to severe diseases, and patients of 50–64 classified in the group of an increased risk of falls should be subject to the multifactorial falls risk assessment (MFRA) and to properly tailored procedures (23, 24), in order to solve, improve, or manage their individual fall risk during their hospitalization.

Many researchers point to age as a key fall risk factor (9–11, 25). Epidemiological studies indicate that the risk of the development of PAD, AAA, and CAD increases together with age (1–3). In our study, the median age in the group with a moderate-to-high risk of falls is substantially higher than in the group with a low risk of falls (71 vs. 68 years of age), which indicates that this is a group of older adult people, and chronic diseases and organ-related changes resulting from aging increase the risk of falls. The guidelines recommend that in hospital, persons of over 65 should be included in the fall risk group (6).

Evidence confirming a relation between female gender and an increased risk of falls is inconsistent (26–28), and our study confirmed that women are subject to an increased risk of falls. Other studies also identified a relation between female gender and falls (29–31). The difference is explained with physiological characteristics and the structure of bones and muscles, as well as hormonal changes related to menopause (29–31).

Arterial hypertension is a serious global health challenge that is very frequent throughout the world. This is an avoidable risk factor of various chronic diseases (32). The literature provides for a confirmed relation between a high risk of falls and hospitalization, a surgical injury and such coexistent diseases as diabetes, arterial hypertension, visual impairment, dizziness, and fear of falling (33, 34). There are many “side effects” of arterial hypertension, which are particularly important in the case of older adult people, including a drop in physical fitness (“worse functioning”) and disability, as well as the frequency of falls and traumatic fractures (35).

The capacity for performing basic daily living activities is a central aspect of quality of life related to health, and a key prognostic factor of hospitalization or surgeries. In this analysis, limitations in daily living activities (ADLs) are considered as a fall risk factor. Other researchers also stated that older adult people that are exposed to the risk of falls are more dependent in their daily living activities and their quality of life is lower (36–39).

In addition, in our study, variables, important only in terms of simple regression, that increase the risk of falls include pain, the number of medicines taken, and the presence of ulceration and balance disorders.

Polypragmasia is defined as a patient taking at least five medicines at once. This has a negative impact in particular on older adult people (40–42). The study indicates that polypragmasia is common and up to 64.6% of patients with a moderate-to-high risk of falls have taken more than 5 medicines at once. Patients have therapy based on many medicines, which is connected with the existence of a great number of coexistent diseases in this group, including arterial hypertension, diabetes, cardiac failure, atherosclerosis, chronic obstructive pulmonary disease, and the age of patients. Polypragmasia contributes to undesired consequences, including balance disorders and an increased risk of falls. The risk of falls increases together with the number of medicines taken by patients and is dependent on the type of medicines. Medicines whose side effects include falls are, among others, sedatives, hypnotic medications, anticholinergic medicines, antihypertensive medicines, antidepressants, and antidiabetics (43).

Epidemiological studies indicate that physical balance disorders in older adult people are common and falls are the most dangerous phenomenon accompanying such disorders. The purpose of the study conducted by Gardner et al. was to discover whether PAD patients have balance disorders and fall more often than the control non-PAD group. They examined 367 PAD patients and 458 non-PAD controls. People with PAD had balance disorders and were more likely to fall, which was connected with the function of walking and daily physical activities (44). The outcome of the study conducted by Suominen et al. also indicated that PAD patients of over 65 years of age had more incidents of balance disorders, constituting a well-known factor of the risk of falls and disability among older adult people (45). Similarly, to our study, Carpenter et al. (46) confirmed that ulceration is an independent factor increasing the risk of falls.

Pain is a recognized fall risk factor. In this study, the intensity of pain was assessed in accordance with the NRS scale. In the study conducted by Li et al. (47), it was confirmed that people suffering from pain were 73% more exposed to falls than those that did not feel pain. The study of Muhammad et al. (48) also provides evidence that pain is a fall risk factor. In addition, the meta-analysis conducted by Stubbs et al. (49) indicated that in the case of older adult people that reported pain, the risk of falls was 56% greater than in the case of people that did not feel pain. The studies conducted in cardiac surgery and vascular surgery may help to improve nursing practices, and the quality of patient care in terms of daily activities or nursing plans, given the patient’s health (50).

## 5 Strengths and limitations

The study has certain limitations. Firstly, the data were collected from patients undergoing vascular procedures at one hospital. Therefore, the results cannot be generalized in terms of a greater population. The risk was assessed at check-in. In turn, as the risk of falls in patients changes continuously during hospitalization, given their health and environment, the risk assessment should be systematically repeated.

## 6 Conclusion

Age, female gender, hypertension, and the Barthel Index were identified as potential factors associated with a moderate-to-high risk of future falls in patients scheduled for vascular surgeries.

## 7 Implications for clinical practice

Although the data in the project are limited, involving patients and their families in care, planning is likely to contribute to reducing the number of falls of patients with a “moderate and high” risk. It is necessary to conduct further studies to include an assessment of patients’ life situations and functional fitness. To optimize patient care, multidisciplinary teams should take into account the support and education needs of patients. An integrated care and support program defined at check-in would reduce the number of falls.

## Data availability statement

The original contributions presented in the study are included in the article/supplementary material; further inquiries can be directed to the corresponding author.

## Ethics statement

The research was approved by the Independent Bioethical Committee at the Medical University of Gdańsk, number NKBBN/348/2022. The studies were conducted in accordance with the local legislation and institutional requirements. The participants provided their written informed consent to participate in this study.

## Author contributions

RP: Conceptualization, Methodology, Visualization, Writing – original draft, Writing – review & editing. WM-D: Visualization, Writing – original draft, Writing – review & editing. LT: Formal Analysis, Visualization, Writing – original draft, Writing – review & editing.

## References

[1] Szymański FM. Diagnosis and pharmacotherapy of patients with peripheral artery disease: what should we remember in everyday practice? Choroby Serca Naczyń. (2014) 3:152–8.

[2] Wanhainen A Verzini F van Herzeele I Allaire E Bown M Cohnert T. European Society for Vascular Surgery (ESVS) 2019 clinical practice guidelines on the management of abdominal aorto-iliac artery aneurysms. Eur J Vasc Endovasc Surg. (2019) 57:8–93. doi: 10.1016/j.ejvs.2018.09.020, PMID: 30528142

[3] DiGiacomo M Prichard R Allida S Delbaere K Omari A Inglis SC. Multifaceted needs of individuals living with peripheral arterial disease: a qualitative study. Chronic Illn. (2022) 18:562–73. doi: 10.1177/1742395321999450, PMID: 33673738

[4] Whipple MO Schorr EN Talley KMC Lindquist R Bronas UG Treat-Jacobson D. A mixed methods study of perceived barriers to physical activity, geriatric syndromes, and physical activity levels among older adults with peripheral artery disease and diabetes. J Vasc Nurs. (2019) 37:91–105. doi: 10.1016/j.jvn.2019.02.001, PMID: 31155168 PMC6556121

[5] Violence and Injury Prevention and Disability (VIP). (2023). Available at: https://www.who.int/news-room/fact-sheets/detail/falls (Accessed June 20, 2023).

[6] National Institute for Health and Care Excellence. (2023). Available at: http://www.nice.org.uk/ (Accessed June 20, 2023).

[7] Graafmans WC Ooms ME Hofstee HM Bezemer PD Bouter LM Lips P. Falls in the elderly: a prospective study of risk factors and risk profiles. Am J Epidemiol. (1996) 143:1129–36. doi: 10.1093/oxfordjournals.aje.a008690 8633602

[8] Ganz DA Bao Y Shekelle PG Rubenstein LZ. Will my patient fall? JAMA. (2007) 297:77–86. doi: 10.1001/jama.297.1.77, PMID: 17200478

[9] Cigolle CT Ha J Min LC Lee PG Gure TR Alexander NB. The epidemiologic data on falls, 1998–2010: more older Americans report FallingThe epidemiologic data on falls, 1998-2010 letters. JAMA Intern Med. (2015) 175:443–5. doi: 10.1001/jamainternmed.2014.7533 25599461 PMC10627480

[10] Sousa LM Marques-Vieira CM Caldevilla MN Henriques CM Severino SS Caldeira SM. Risk for falls among community-dwelling older people: systematic literature review. Rev Gaucha Enferm. (2017) 37:e55030. doi: 10.1590/1983-1447.2016.04.55030, PMID: 28273251

[11] Lukaszyk C Harvey L Sherrington C Keay L Tiedemann A Coombes J. Risk factors, incidence, consequences and prevention strategies for falls and fall-injury within older indigenous populations: a systematic review. Aust N Z J Public Health. (2016) 40:564–8. doi: 10.1111/1753-6405.12585, PMID: 27774702

[12] Ungar A Mussi C Ceccofiglio A Bellelli G Nicosia F Bo M. Etiology of syncope and unexplained falls in elderly adults with dementia: syncope and dementia (SYD) study. J Am Geriatr Soc. (2016) 64:1567–73. doi: 10.1111/jgs.14225, PMID: 27351866

[13] Galizia G Langellotto A Cacciatore F Mazzella F Testa G Della-Morte D. Association between nocturia and falls-related long-term mortality risk in the elderly. J Am Med Dir Assoc. (2012) 13:640–4. doi: 10.1016/j.jamda.2012.05.016 22763143

[14] Nakagawa H Niu K Hozawa A Ikeda Y Kaiho Y Ohmori-Matsuda K. Impact of nocturia on bone fracture and mortality in older individuals: a Japanese longitudinal cohort study. J Urol. (2010) 184:1413–8. doi: 10.1016/j.juro.2010.05.093 20727545

[15] Stevens JA Phelan EA. Development of STEADI: a fall prevention resource for health care providers. Health Promot Pract. (2013) 14:706–14. doi: 10.1177/1524839912463576, PMID: 23159993 PMC4707651

[16] Stevenson DG Studdert DM. The rise of nursing home litigation: findings from a national survey of attorneys. Health Affairs. (2003) 22:219–29. doi: 10.1377/hlthaff.22.2.219, PMID: 12674425

[17] Chou YJ Kuo HJ Shun SC. Cancer Prehabilitation programs and their effects on quality of life. Oncol Nurs Forum. (2018) 45:726–36. doi: 10.1188/18.ONF.726-736 30339146

[18] Vandenbroucke JP von Elm E Altman DG Gøtzsche PC Mulrow CD Pocock SJ. STROBE initiative. Strengthening the reporting of observational studies in epidemiology (STROBE): explanation and elaboration. Int J Surg. (2014) 12:1500–24. doi: 10.1016/j.ijsu.2014.07.014, PMID: 25046751

[19] Podsiadlo D Richardson S. The timed “up & go”: a test of basic functional mobility for frail elderly persons. J Am Geriatr Soc. (1991) 39:142–8. doi: 10.1111/j.1532-5415.1991.tb01616.x 1991946

[20] Shafshak TS Elnemr R. The visual analogue scale versus numerical rating scale in measuring pain severity and predicting disability in low Back pain. J Clin Rheumatol. (2021) 27:282–5. doi: 10.1097/RHU.0000000000001320, PMID: 31985722

[21] Mahoney F Barthel D. Functional evaluation: the Barthel index. Md State Med J. (1965) 14:61–5. PMID: 14258950

[22] Stanisz A. Przystępny kurs Statystyki z Zastosowaniem STATISTICA PL na Przykładach z Medycyny, tom 2, Modele Liniowe i Nieliniowe. Kraków: Wydawnictwo StatSoft Polska (2000).

[23] Alvarado N McVey L Wright J Healey F Dowding D Cheong VL. Exploring variation in implementation of multifactorial falls risk assessment and tailored interventions: a realist review. BMC Geriatr. (2023) 23:381. doi: 10.1186/s12877-023-04045-3, PMID: 37344760 PMC10286425

[24] National Institute for Health and Clinical Excellence. Falls in Older People: Assessing Risk and Prevention: Clinical Guideline. London: NICE (2013). 31869045

[25] Mazur K Wilczyński K Szewieczek J. Geriatric falls in the context of a hospital fall prevention program: delirium, low body mass index, and other risk factors. Clin Interv Aging. (2016) 11:1253–61. doi: 10.2147/CIA.S115755, PMID: 27695303 PMC5027952

[26] Oneil CA Krauss MJ Bettale J Kessels A Costantinou E Dunagan WC. Medications and patient characteristics associated with falling in the hospital. J Patient Saf. (2018) 14:27–33. doi: 10.1097/PTS.0000000000000163, PMID: 25782559 PMC4573384

[27] Halfon P Eggli Y Van Melle G Vagnair A. Risk of falls for hospitalized patients: a predictive model based on routinely available data. J Clin Epidemiol. (2001) 54:1258–66. doi: 10.1016/S0895-4356(01)00406-1 11750195

[28] Brand CA Sundararajan V. A 10-year cohort study of the burden and risk of in-hospital falls and fractures using routinely collected hospital data. Qual Saf Health Care. (2010) 19:e51. doi: 10.1136/qshc.2009.038273 20558479

[29] Ambrose AF Paul G Hausdorff JM. Risk factors for falls among older adults: a review of the literature. Maturitas. (2013) 75:51–61. doi: 10.1016/j.maturitas.2013.02.009 23523272

[30] Smith AA Silva AO Rodrigues RA Moreira MA Nogueira JA Tura LF. Assessment of risk of falls in elderly living at home. Rev Lat Am Enfermagem. (2017) 25:e2754. doi: 10.1590/1518-8345.0671.2754, PMID: 28403333 PMC5396481

[31] Chang VC Do MT. Risk factors for falls among seniors: implications of gender. Am J Epidemiol. (2015) 181:521–31. doi: 10.1093/aje/kwu268, PMID: 25700887

[32] Li L Gan Y Zhou X Jiang H Zhao Y Tian Q. Insomnia and the risk of hypertension: a meta-analysis of prospective cohort studies. Sleep Med Rev. (2021) 56:101403. doi: 10.1016/j.smrv.2020.101403 33360604

[33] Bittencourt VLL Graube SL Stumm EMF Battisti IDE Loro MM. Winkelmann ER. Factors associated with the risk of falls in hospitalized adult patients. Rev Esc Enferm USP. (2017) 51:e03237. doi: 10.1590/S1980-220X2016037403237 28746559

[34] Neto AAH Patrício ACFA Ferreira MAM Rodrigues BFL Santos TDD Rodrigues TDB. Falls in institutionalized older adults: risks, consequences and antecedents. Rev Bras Enferm. (2017) 70:719–25. doi: 10.1590/0034-7167-2017-0107, PMID: 28793100

[35] Buford TW. Hypertension and aging. Ageing Res Rev. (2016) 26:96–111. doi: 10.1016/j.arr.2016.01.007, PMID: 26835847 PMC4768730

[36] Çinarli T Koç Z. Fear and risk of falling, activities of daily living, and quality of life: assessment when older adults receive emergency department care. Nurs Res. (2017) 66:330–5. doi: 10.1097/NNR.0000000000000227 28654570

[37] Byun M Kim J Kim JE. Physical and psychological factors contributing to incidental falls in older adults who perceive themselves as unhealthy: a cross-sectional study. Int J Environ Res Public Health. (2021) 18:1–12. doi: 10.3390/ijerph18073738, PMID: 33918455 PMC8038270

[38] Velegraki M Ioannou P Tsioutis C Persynaki GS Pediaditis E Koutserimpas C. Age, comorbidities and fear of fall: mortality predictors associated with fall-related fractures. Maedica. (2020) 15:18–23. doi: 10.26574/maedica.2020.15.1.18, PMID: 32419856 PMC7221284

[39] Abaraogu UO Dall PM Brittenden J Stuart W Tew GA Godwin J. Efficacy and feasibility of pain management and patient education for physical activity in intermittent claudication (PrEPAID): protocol for a randomised controlled trial. Trials. (2019) 20:1–12. doi: 10.1186/s13063-019-3307-6 PMC6469131 30992033

[40] Jankowska-Polańska B Uchmanowicz I. Polypharmacy, comorbidities and falls in elderly patients with chronic heart failure. Geriatria. (2014) 8:1–12.

[41] Gnjidic D Hilmer SN Blyth FM Naganathan V Waite L Seibel MJ. Polypharmacy cutoff and outcomes: five or more medicines were used to identify community-dwelling older men at risk of different adverse outcomes. J Clin Epidemiol. (2012) 65:989–95. doi: 10.1016/j.jclinepi.2012.02.018, PMID: 22742913

[42] Chou J Tong M Brandt NJ. Combating polypharmacy through deprescribing potentially inappropriate medications. J Gerontol Nurs. (2019) 45:9–15. doi: 10.3928/00989134-20190102-01 30653232

[43] Hilmer SN Gnjidic D. The effects of polypharmacy in older adults. Clin Pharmacol Ther. (2009) 85:86–8. doi: 10.1038/clpt.2008.224 19037203

[44] Gardner AW Montgomery PS. Impaired balance and higher prevalence of falls in subjects with intermittent claudication. J Gerontol A Biol Sci Med Sci. (2001) 56:M454–8. doi: 10.1093/gerona/56.7.m454, PMID: 11445605

[45] Suominen V Salenius J Sainio P Reunanen A Rantanen T. Peripheral arterial disease, diabetes and postural balance among elderly Finns: a population-based study. Aging Clin Exp Res. (2008) 20:540–6. doi: 10.1007/BF03324882, PMID: 19179838

[46] Carpenter CR Scheatzle MD D’Antonio JA Ricci PT Coben JH. Identification of fall risk factors in older adult emergency department patients. Acad Emerg Med. (2009) 16:211–9. doi: 10.1111/j.1553-2712.2009.00351.x 19281493

[47] Li W Gamber M Han J Sun W Yu T. The association between pain and fall among middle-aged and older Chinese. Pain Manag Nurs. (2021) 22:343–8. doi: 10.1016/j.pmn.2020.10.004, PMID: 33272831

[48] Muhammad T Maurya P Selvamani Y Kelekar U. Mediation of pain in the association of sleep problems with falls among older adults in India. Sci Rep. (2023) 13:221. doi: 10.1038/s41598-022-27010-3, PMID: 36604470 PMC9816101

[49] Stubbs B Binnekade T Eggermont L Sepehry AA Patchay S Schofield P. Pain and the risk for falls in community-dwelling older adults: systematic review and meta-analysis. Arch Phys Med Rehabil. (2014) 95:175–187.e9. doi: 10.1016/j.apmr.2013.08.241, PMID: 24036161

[50] Brčina A Civka K Habeković R Krupa S Ljubas A Mędrzycka-Dąbrowska W. Prevalence of postoperative atrial fibrillation and impact to nursing practice-a cross sectional study. Med Sci. (2023) 11:1–12. doi: 10.3390/medsci11010022, PMID: 36976530 PMC10056994

